# Red blood cell distribution width to albumin ratio as a novel predictor for mortality in chronic obstructive pulmonary disease patients: Results from the cohort study of NHANES, 1999–2018

**DOI:** 10.1371/journal.pone.0319869

**Published:** 2025-04-01

**Authors:** Guoxin Zhang, Beizheng Xu, Xiaoyun Zhao

**Affiliations:** 1 Department of Respiratory and Critical Care Medicine, Tianjin University Chest Hospital, Thoracic Clinical College of Tianjin Medical University, Tianjin Chest Hospital, Tianjin, China; 2 Tianjin Key Laboratory of Ionic-Molecular Function of Cardiovascular Disease, Department of Cardiology, Tianjin Institute of Cardiology, Second Hospital of Tianjin Medical University, Tianjin, China; Mansoura University Faculty of Veterinary Medicine, EGYPT

## Abstract

**Objectives:**

To investigate the association of red blood cell distribution width (RDW) to albumin ratio (RAR) with mortality in chronic obstructive pulmonary disease (COPD) patients.

**Methods:**

We selected 1,652 patients with COPD from the National Health and Nutrition Examination Survey (NHANES) 1999-2018, who were categorized into four groups according to the RAR quartiles. Kaplan-Meier curves, restricted cubic splines and the Cox proportional hazard model were used to evaluate the associations between RAR and all-cause mortality and chronic lower respiratory disease (CLRD) mortality in the COPD patients. Subgroup analyses were performed to check the interaction of the different characteristics.

**Results:**

There were 640 deaths during follow-up, of which, 145 were from CLRD. Kaplan-Meier curves indicated COPD patients with higher RAR had significantly increased all-cause mortality and CLRD mortality. Multivariate Cox regression analyses showed HR of Q4 RAR was 2.88 (95% CI 2.18 - 3.81, *p* < 0.0001) for all cause-mortality and 3.39 (95% CI 1.76 - 6.53, *p* < 0.001) for CLRD mortality, compared with Q1 RAR. Restricted cubic splines analysis indicated a dose-response between RAR and risk of all-cause and CLRD mortality (*p* for non-linearity < 0.001).

**Conclusion:**

RAR had an independent association with all-cause mortality, especially CLRD mortality, in COPD patients. RAR has potential as a novel and promising predictor to identify COPD individuals with high mortality risk.

## Introduction

Chronic obstructive pulmonary disease (COPD) is a heterogeneous lung condition characterized by persistent, often progressive, airflow obstruction, leading to repeated worsening of respiratory symptoms [1,2]. COPD is the third leading cause of death worldwide, causing 3.23 million deaths in 2019, and 90% of these deaths occurred in low- and middle-income countries [3,4]. The prevalence and burden of COPD are projected to increase over the coming decades due to a combination of continued exposure to risk factors and aging of the global population [5]. COPD often co-exists with comorbidities (cardiovascular diseases, heart failure, arrhythmias, osteoporosis, anaemia, polycythaemia, and frailty etc.) that may have a significant impact on prognosis [1]. There is an urgent need for effective factors to predict COPD mortality.

Red blood cell distribution width (RDW), a simple and inexpensive parameter traditionally used in laboratory haematology, can reflect the degree of heterogeneity of erythrocyte volume and identify differential diagnosis of anaemia [6]. Recent evidence indicates that elevated RDW is associated with poor outcomes of aortic aneurysm [7], acute pancreatitis [8], postoperative hepatocellular carcinoma [9], myocardial infarction [10] and heart failure [11]. Serum albumin, the most abundant protein in plasma, plays a major role in maintaining plasma osmotic pressure and transferring bioactive substances. There is evidence to indicate that reduction of albumin is associated with infection, systemic inflammation, malnutrition and frailty [12–15]. The RDW to albumin ratio (RAR) is a newly derived marker that is considered to be an indicator of inflammation and malnutrition. Recent data support the potential of RAR in predicting adverse outcomes, such as, increased risk mortality of acute myocardial infarction [16,17], greater likelihood of progression to diabetic kidney disease [18], increased in-hospital mortality and 1-year mortality of pulmonary embolism [19], increased short- and long-term mortality of heart failure [20], and increased all-cause mortality in cancer [21].

Inflammation, oxidative stress, and aging are important potential mechanism of COPD occurrence and progression [1]. Both serum albumin and RDW are indicated to related to inflammation and oxidative stress, as well as aging [22–29]. Undoubtedly, RAR, a derived marker from RDW and albumin, have the potential to amplify the pathophysiological processes. Previous studies have focused on the relationship between RAR and COPD mortality. However, these studies involved COPD patients hospitalised or admitted to the intensive care unit (ICU) because of acute exacerbation [30–32]. Therefore, it was not possible to establish whether RAR was associated with mortality in COPD patients who were not in hospital or did not have acute exacerbation. The National Health and Nutrition Examination Survey (NHANES) database includes a diverse, representative population and therefore comprises accurate and detailed data. NHANES provides a unique opportunity to resolve the issue that hospitalised COPD patients cannot represent the overall COPD population, with or without acute exacerbation. This study aimed to investigate the association between RAR and mortality of the overall population of COPD patients.

## Material and methods

### Study design and subjects

NHANES is an annual survey conducted by the National Centre for Health Statistics of the US Centres for Disease Control and Prevention. NHANES aims to collect data from a representative sample of the non-institutionalised American population. NHANES uses a stratified, multistage probability sampling strategy to recruit a cross-section of the American population. Data are obtained through structured household interviews, health examinations at mobile examination centres, and laboratory sample analyses. Trained interviewers collect the necessary data during these interviews.

We conducted a secondary analysis of de-identified and publicly available NHANES data. Therefore, ethics approval from an institutional review board and informed consent from participants were not required. Detailed statistical data can be found on the NHANES website (https://www.cdc.gov/nchs/nhanes/). Our study complied with the STROBE guidelines for reporting observational epidemiological studies.

**Fig 1:**
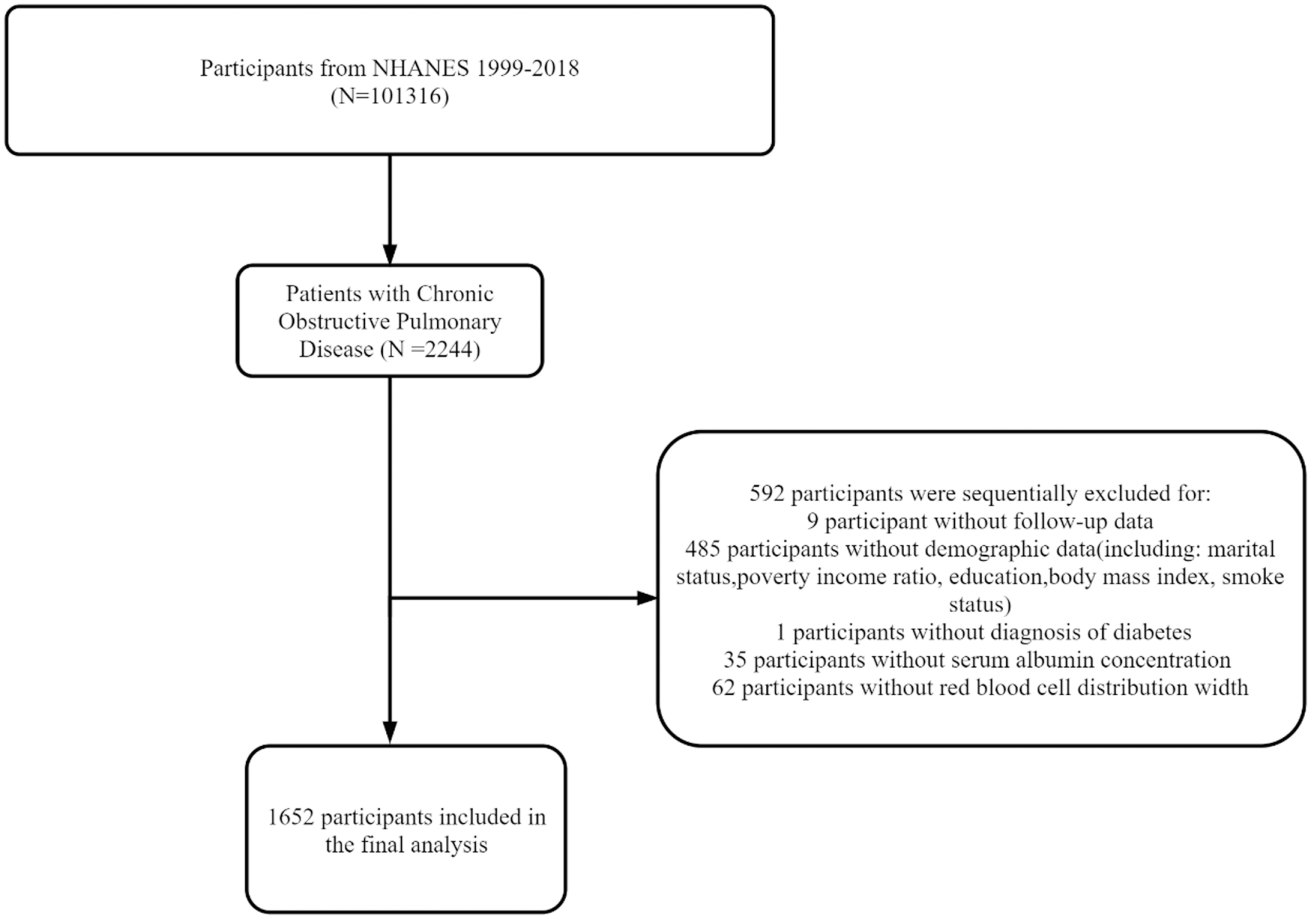
Flow diagram for selection of study participants. NHANES, National Health and Nutrition Examination Survey.

From 1999 to 2018, NHANES included 101,316 individuals, and 2,244 COPD patients were selected. The following patients were excluded from our study: nine without follow-up data; 485 with incomplete basic information (including marital status, poverty income ratio [PIR], education level, body mass index [BMI], and smoking status); 62 with missing RDW data; 35 with missing serum albumin data; and one with missing diabetes mellitus (DM) data. The final sample consisted of 1,652 participants, representing 6,157,715 people in the USA. The screening process is detailed in Fig 1.

### COPD diagnosis

As reported in GOLD 2024, one of the key criteria for diagnosing COPD is the presence of non-fully reversible airflow obstruction (FEV1/FVC < 0.7 post-bronchodilation) measured by spirometry [1]. However, numerous previous studies using the NHANES data have also employed additional methods, such as a positive response to a questionnaire, specific medication history, and FEV1/FVC ratio < 0.7, to identify COPD patients. This approach has been widely accepted and successfully implemented. [2,25,33–38]. Similarly, in this study, COPD was confirmed if participants responded positively to questions about physician-diagnosed COPD, emphysema or chronic bronchitis. Participants who answered “yes” were classified as COPD-positive, while those who answered “no” were classified as COPD-negative. Patients with an FEV1/FVC ratio <0.7 on pulmonary function tests were also classified as COPD-positive. The COPD group also included patients aged ≥40 years with a smoking history or chronic bronchitis, who were using specific medications such as selective phosphodiesterase-4 inhibitors, mast cell stabilizers, leukotriene modulators and inhaled corticosteroids

### RAR measurement

Serum albumin concentration was determined using the bromocresol purple method. RDW (percentage) was measured in peripheral blood samples using a Coulter analyser at the mobile examination centres.

### Mortality status

Follow-up and mortality data were obtained by linking NHANES data to the National Death Index records up to December 31, 2019. The underlying cause of death was determined using codes from the 10th revision of the International Classification of Diseases. All-cause mortality refers to death from any cause, including heart disease, Alzheimer’s disease, chronic lower respiratory disease (CLRD), influenza and pneumonia, malignant neoplasms, accidents (unintentional injuries), nephritis, nephrotic syndrome and nephrosis, cerebrovascular disease, and DM. CLRD mortality was defined as death from asthma (Code J45-J46), emphysema (J43), bronchitis (chronic or other) (J40-J42) or other CLRD (J44 and J47).

### Covariates

This study considered various demographic factors as confounders, including: age; sex (male and female); race and ethnicity (non-Hispanic white, non-Hispanic black, Mexican American, and others); marital status (married/living with partner, never married, and widowed/divorced/separated); PIR [classified as low income (<1.30), middle income (1.30-3.49), and high income (≥3.50)]; and educational level (below high school, high school, and college or above). Smoking status was categorized as never smokers (<100 cigarettes in a lifetime); current smokers (≥100 cigarettes in a lifetime); and former smokers (≥100 cigarettes in a lifetime and having quit smoking). Alcohol consumption was classified as never, former, mild, moderate, and heavy, specifically defined as: heavy (≥3 drinks per day for females; ≥ 4 drinks per day for males or binge drinking on ≥ 5 days per month); moderate (≥2 drinks per day for females; ≥ 3 drinks per day for males or binge drinking ≥ 2 days per month); and mild (not meeting the aforementioned criteria). BMI was measured and categorized into < 25.0, 25.0-29.9 and > 29.9 kg/m^2^. Diagnosis of CVD (coronary heart disease, congestive heart failure, heart attack, stroke or angina) was obtained through standardized questionnaires. Diagnosis of DM was made if patients reported a physician's diagnosis of DM, used insulin or took oral hypoglycaemic drugs.

### Statistical analysis

By considering the complex sampling design of NHANES, all analyses in this study accounted for sample weights, clustering and stratification to generate nationally representative estimates. Detailed descriptions of the weighting methods are available on the NHANES website. First, categorical variables were expressed as weighted percentages, while continuous variables were expressed as weighted means with corresponding confidence intervals (CIs). Second, weighted Cox proportional hazards regression models were used to calculate hazard ratios (HRs) and 95% CIs to examine the association between RAR and all-cause/CLDR-specific mortality risk in COPD patients. Model 1 was used for unadjusted analysis; Model 2 included adjustments for age (continuous), sex (male, female), race and ethnicity (non-Hispanic black, Non-Hispanic white, Mexican American, other); and Model 3 included adjustments for age (continuous), sex (male, female), race and ethnicity (non-Hispanic black, non-Hispanic white, Mexican American, other), marital status (married/living with partner, widowed/divorced/separated and never married), PIR (classified as low income [<1.30], middle income [1.30–3.49], and high income [≥3.50]), and educational level ( <high school, high school, and college or above), BMI (<25.0, 25.0–29.9, > 29.9), smoking status (never, former, now), alcohol consumption (never, former, mild, moderate, heavy), DM status (yes, no), and CVD status (yes, no). Third, stratified analyses were conducted based on age strata, sex, race and ethnicity, educational level, BMI, PIR, marital status, smoking status, DM status and CVD status to examine the impact of demographic differences on the results. The *p* value for interaction between RAR and stratified factors was used to estimate the significance of the interaction. Fourth, restricted cubic spline (RCS) regression was used to test the potential nonlinear relationship between RAR and all-cause and CLDR-related mortality in COPD patients. In many fields, relationships are rarely perfectly linear. RCS has the ability to capture complex, non-linear trends in the data. So that, a more accurate fit to the data can be achieved, as the model is no longer constrained by the assumption of linearity. Likelihood ratio tests were used to detect nonlinear relationships. Kaplan-Meier survival analysis was performed to plot the association between different RAR and all-cause/CLDR mortality. All analyses were performed using R version 4.3.3. *p* < 0.05 was considered statistically significant.

## Results

### Baseline characteristics of the patients

This study included a total of 1,652 COPD patients [711 female (48.36%), mean age (standard error) 60.36 (0.41) years]. There were 1,127 (84.52%) non-Hispanic white patients, 256 (6.37%) non-Hispanic black, 87 (6.26%) Mexican American, and 182 (7.67%) classified as others. Baseline characteristics are summarized in Table 1. Participants with higher RAR were more likely to be older, female, non-Hispanic white, married, or cohabiting, with low income, and high BMI. They were less likely to have cardiovascular disease or diabetes. There were no significant differences in educational level, smoking, or alcohol consumption.

**Table 1:** Characteristics by RAR.

Variable	Total	RAR				*p* value
	(N = 1652)	Q1, 2.255–2.932 (N = 417)	Q2, 2.932–3.175 (N = 411)	Q3, 3.175–3.524 (N = 412)	Q4, 3.524–10.216 (N = 411)	
Age	60.36(0.41)	56.95(0.81)	59.67(0.76)	62.72(0.84)	63.82(0.71)	<0.0001
**Age strata**						<0.0001
−39	4.93(0.01)	9.23(2.10)	4.23(1.16)	3.63(1.12)	0.88(0.38)	
40–59	41.91(0.03)	50.60(3.14)	45.26(3.30)	32.50(3.31)	34.45(3.36)	
60-	53.16(0.02)	40.17(2.94)	50.51(3.32)	63.87(3.41)	64.67(3.33)	
**Sex**						<0.001
Female	49.29(0.03)	37.81(2.97)	52.75(3.29)	55.48(3.63)	55.24(3.57)	
Male	50.71(0.03)	62.19(2.97)	47.25(3.29)	44.52(3.63)	44.76(3.57)	
**Race and ethnicity**						<0.0001
Non-Hispanic White	84.52(0.05)	89.42(1.97)	88.25(1.87)	79.84(1.96)	77.27(2.42)	
Non-Hispanic Black	6.37(0.01)	2.43(0.57)	4.00(0.76)	10.02(1.34)	11.49(1.50)	
Mexican American	1.44(0.00)	1.01(0.29)	1.02(0.25)	2.03(0.61)	2.00(0.55)	
Other	7.67(0.01)	7.14(1.72)	6.73(1.65)	8.11(1.38)	9.24(1.91)	
**Education levels**						0.59
<high school	24.40(0.02)	22.32(3.09)	24.45(2.72)	24.01(2.83)	27.77(2.45)	
high school	25.52(0.02)	23.42(2.32)	27.14(3.00)	28.26(3.39)	23.68(2.64)	
college or above	50.08(0.03)	54.26(3.35)	48.40(3.40)	47.74(3.39)	48.55(2.87)	
**Marital status**						0.003
Married/Living with Partner	63.23(0.04)	68.84(2.83)	66.58(2.81)	57.30(3.09)	56.70(3.23)	
Widowed/Divorced/Separated	30.81(0.02)	24.49(2.49)	27.38(2.41)	37.12(3.20)	38.08(3.15)	
Never married	5.96(0.01)	6.67(1.45)	6.04(1.30)	5.58(1.18)	5.22(0.92)	
**PIR**						<0.0001
Low income	27.34(0.02)	17.85(2.29)	26.16(2.95)	33.10(4.03)	36.85(2.95)	
Middle income	36.38(0.02)	34.13(2.83)	34.67(3.23)	43.53(3.31)	34.64(3.34)	
High income	36.28(0.03)	48.03(3.60)	39.17(3.40)	23.37(3.27)	28.51(3.84)	
**BMI**	29.27(0.26)	27.01(0.34)	29.11(0.45)	29.82(0.48)	32.22(0.77)	<0.0001
**Smoke status**						0.91
Never	16.55(0.01)	17.62(2.23)	16.42(2.31)	13.85(2.31)	17.90(2.94)	
Former	47.07(0.03)	46.18(2.83)	47.30(3.19)	47.14(3.94)	48.01(3.13)	
Now	36.38(0.03)	36.20(2.92)	36.28(3.01)	39.01(3.75)	34.09(3.71)	
**Alcohol use status**						0.1
Never	6.17(0.01)	5.86(1.46)	4.97(1.28)	8.84(1.50)	5.44(1.44)	
Former	27.92(0.02)	22.83(3.03)	28.90(2.95)	25.00(2.39)	37.03(3.02)	
Mild	34.42(0.02)	36.97(3.58)	30.67(2.74)	37.20(3.03)	32.77(3.47)	
Moderate	15.28(0.01)	15.80(2.14)	17.29(2.83)	15.74(2.40)	11.45(3.13)	
Heavy	16.22(0.02)	18.55(2.77)	18.17(2.64)	13.22(2.51)	13.31(2.69)	
**CVD**						<0.0001
Yes	27.78(0.02)	15.32(2.01)	24.94(2.82)	33.01(3.11)	44.35(3.32)	
No	72.22(0.04)	84.68(2.01)	75.06(2.82)	66.99(3.11)	55.65(3.32)	
**DM**						<0.0001
Yes	23.31(0.02)	12.15(1.68)	20.44(2.57)	29.36(2.79)	37.13(3.05)	
No	76.69(0.04)	87.85(1.68)	79.56(2.57)	70.64(2.79)	62.87(3.05)	

Values were presented as number (percentage) or weighted means (standard errors).

BMI, body mass index; CVD cardiovascular disease; DM diabetes mellitus; PIR poverty income ratio; RAR ratio of red blood cell distribution width to albumin concentration.

Q1 (2.255–2.932), Q2 (2.932–3.175), Q3 (3.175–3.524), Q4 (3.524–10.216).

### Association between RAR and mortality in COPD patients

**Fig 2:**
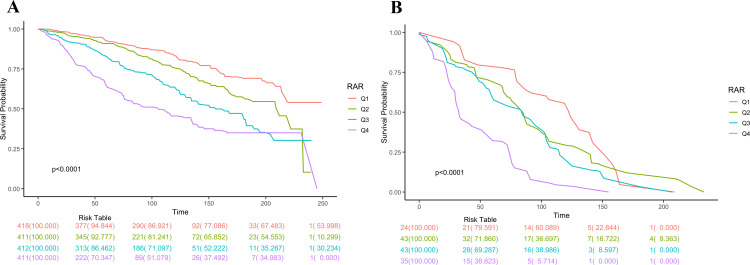
Cumulative incidence of all-cause (A) and CLRD (B) mortality in the four grades of RAR during the follow-up period. CLRD, chronic lower respiratory diseases; Q1–Q4, quartiles 1–4; RAR, red blood distribution width to albumin ratio.

The median follow-up time was 94 months, during which, 640 deaths were recorded, of which, 145 were attributed to CLRD. Kaplan-Meier curves showed that COPD patients with higher RAR had significantly increased all-cause mortality (p < 0.0001). As illustrated in Fig 2A, compared with RAR Q1 group, the survival probability was much lower in RAR Q4 group at every time point. Similar patterns were observed for CLRD mortality (Fig 2B). Cox proportional hazards model confirmed that patients with higher RAR had significantly increased mortality risk in all models. Specifically, in the multivariable adjusted Model 3, higher levels of RAR were associated with increased all-cause mortality (HR 1.69, 95% CI 1.48–1.93) as well as CLRD mortality (HR 3.64, 95% CI 2.38–5.58). With Q1 (2.255–2.932) as reference: Q2 (2.932–3.175) HR 1.25, 95% CI 0.98–1.61; Q3 (3.175–3.524) HR 1.70, 95% CI 1.29–2.25; and Q4 (3.524–10.216) HR 2.88 95% CI 2.18–3.81), with a significant increasing trend (*p* for trend < 0.0001). For CLRD mortality, with Q1 (2.255–2.932) as reference: Q2 (2.932–3.175) HR 1.07, 95% CI 0.58–1.95; Q3 (3.175–3.524) HR 1.24, 95% CI 0.65–2.37; and Q4 (3.524–10.216) HR 3.39, 95% CI 1.76–6.53 (*p* for trend = 0.001) (Table 2).

**Table 2:** Crude and adjusted association between RAR and mortality among COPD patients.

Model	RAR	Hazard ratio (95% CI)				*p* for trend
		Q1, 2.255–2.932	Q2, 2.932–3.175	Q3, 3.175–3.524	Q4, 3.524–10.216	
All-cause mortality						
Model 1	1.86(1.58,2.18)	1.000(Reference)	1.51(1.15,1.99)	2.43(1.85,3.19)	4.22(3.14,5.66)	
*p* values	<0.0001		0.003	<0.0001	<0.0001	<0.0001
Model 2	1.79(1.57,2.05)	1.000(Reference)	1.37(1.07,1.75)	1.81(1.34,2.45)	3.31(2.53,4.33)	
*p* values	<0.0001		0.01	<0.001	<0.0001	<0.0001
Model 3	1.69(1.48,1.93)	1.000(Reference)	1.25(0.98,1.61)	1.70(1.29,2.25)	2.88(2.18,3.81)	
*p* values	<0.0001		0.08	<0.001	<0.0001	<0.0001
CLRD mortality						
Model 1	3.59(2.58,4.98)	1.000(Reference)	1.13(0.67,1.91)	1.55(0.98,2.46)	3.55(2.26,5.58)	
*p* values	<0.0001		0.64	0.06	<0.0001	<0.0001
Model 2	3.62(2.53,5.18)	1.000(Reference)	1.11(0.65,1.90)	1.44(0.89,2.35)	3.38(2.23,5.11)	
*p* values	<0.0001		0.71	0.14	<0.0001	<0.0001
Model 3	3.64(2.38, 5.58)	1.000(Reference)	1.07(0.58,1.95)	1.24(0.65,2.37)	3.39(1.76,6.53)	
*p* values	<0.0001		0.83	0.52	<0.001	0.001

Model 1: unadjusted model.

Model 2: adjusted for age (continuous), sex (male, female), race and ethnicity (non-Hispanic black, Non-Hispanic white, Mexican American, other).

Model 3: adjusted for age (continuous), sex (male, female), race and ethnicity (non-Hispanic black, non-Hispanic white, Mexican American, other), marital status (married/living with partner, widowed/divorced/separated and never married), poverty income ratio (classified as low income [<1.30], middle income [1.30–3.49], and high income [≥3.50]), and educational level ( <high school, high school, and college or above), body mass index (<25.0, 25.0–29.9, > 29.9), smoking status (never, former, now), alcohol consumption (never, former, mild, moderate, heavy), diabetes (yes, no), and cardiovascular disease (yes, no).

**Fig 3:**
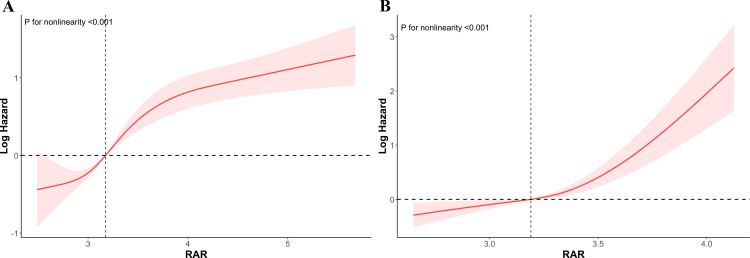
The RCS curve showed a dose response between RAR and all-cause (A)/ CLRD (B) mortality. Adjusted for age, sex, race and ethnicity. RCS, restricted cubic spline; RAR, red blood distribution width to albumin ratio; CLRD, chronic lower respiratory diseases.

The dose-response association between RAR and all-cause and CLRD mortality is shown in Fig 3. The RCS curve indicated a significant nonlinear relationship between RAR and all-cause mortality and CLRD mortality (*p* overall < 0.001, *p* for nonlinearity < 0.001).

### Subgroup analysis

**Fig 4:**
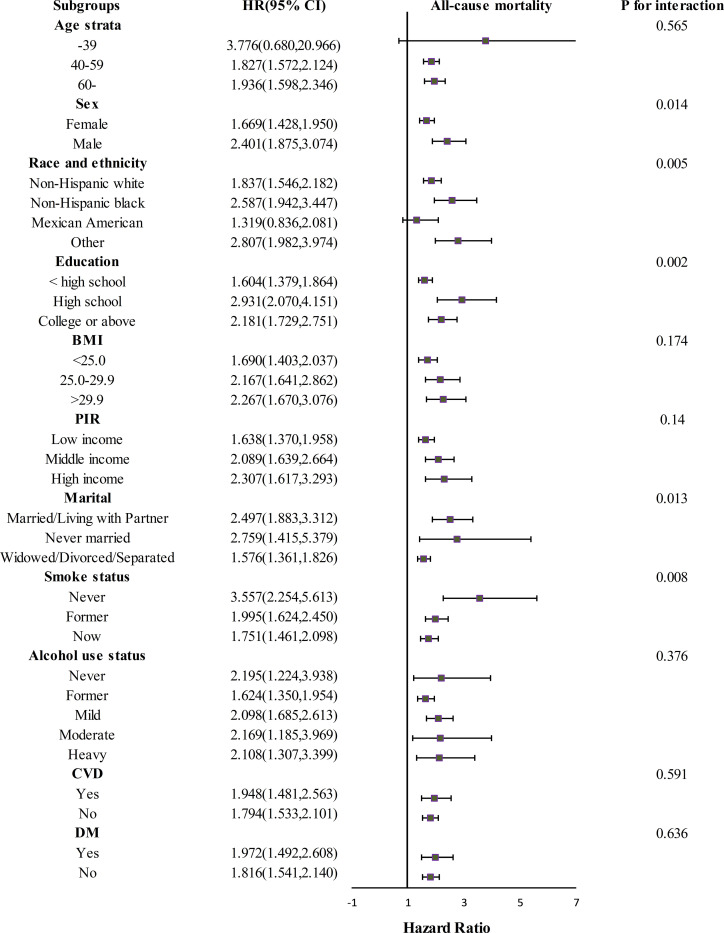
Associations between RAR and all-cause mortality among subgroups. BMI, body mass index; CI, confidence interval; CVD, cardiovascular disease; DM, diabetes mellitus; HR, hazard ratio; PIR, poverty income ratio; RAR, red blood distribution width to albumin ratio.

We further examined interactions and conducted subgroup analyses regarding RAR and mortality in the COPD population. Subgroup analyses were stratified by factors such as age, sex, race/ethnicity, education level, BMI, PIR, marital status, smoking behaviour, alcohol consumption, CVD, and DM. The results indicated interactions in the following subgroups: sex, race and ethnicity, education level, marital status, and smoking status (*p* for interaction < 0.05). Fig 4 shows the HRs and 95% CIs, and the *p* for interaction for each subgroup after stratification.

## Discussion

We utilized the NHANES database to evaluate the relationship between RAR and mortality in 1,652 COPD patients at baseline and 640 deaths during follow-up from 1999 to 2018. Higher RAR was independently associated with increased risk of all-cause and CLRD mortality. When stratified by quartiles, higher quartiles of RAR were associated with greater risk of mortality. With Q1 as a reference, the HR for all-cause mortality in Q4 was 2.88 (95% CI 2.18–3.81), and 3.39 (95% CI 1.76–6.53) for CLRD mortality. RCS analysis also revealed all-cause and CLRD-specific mortality increased in a nonlinear manner as RAR increased. Previous studies involved COPD patients who were hospitalised or admitted to ICU with acute exacerbation. Therefore, those studies could not establish whether RAR was associated with mortality in COPD patients who were not in hospital or did not have acute exacerbation. It is hoped that our results will allow us to use RAR to predict mortality in COPD patients whether or not they are in hospital or have acute exacerbation. Compared with all-cause mortality, RAR had greater value in predicting CLRD mortality. This indicated that RAR had significant prognostic value for mortality risk of COPD patients.

Previous studies have focused on RDW or albumin. Zorlu reported a 14.6% increase in RDW compared with baseline associated with increased risk for early mortality related to acute pulmonary embolism [39]. A study with 27,124 patients showed that HR for incidence of atrial fibrillation was 1.33 for Q4 versus Q1 of RDW [40]. Rahimirad et al. investigated the hospital records of 330 patients and showed that higher RDW on admission was associated with increased in-hospital mortality in patients with acute exacerbation of COPD [30]. Liao et al. reported that serum albumin level was independently inversely associated with incidence of atrial fibrillation in a linear pattern [41]. A meta-analysis showed that elevated levels of serum albumin were associated with reduced risk of vascular outcomes, all-cause mortality, some cancers, and fractures [15].

RDW is positively associated with adverse outcomes, whereas albumin is negatively associated. We suggest that RAR is better than either RDW or albumin alone in predicting adverse outcomes. Zhao et al. were the first, to our knowledge, to divide RDW by albumin to form RAR. Their results from the MIMIC-Ⅲ database suggested that high RAR was associated with stroke-associated infections and mortality in stroke patients [42]. Lu and colleagues’ data supported that increased RAR was independently associated with increased all-cause mortality in cancer patients [21]. Hao and colleagues’ results showed elevated RAR was connected with greater all-cause mortality risk [43]. The association between RAR and adverse outcomes has been found in heart failure, pulmonary embolism, acute myocardial infarction, and acute kidney injury [16–20,44].

There have been previous studies to investigate the connection between RAR and COPD. A prospective study involving 160 inpatients with acute exacerbation of COPD showed that those in need of non-invasive mechanical ventilation or long-term oxygen therapy had higher RAR than those were not [45]. Eraslan et al. conducted a retrospective study involving 58 patients with COPD exacerbation, which revealed an insignificant association between RAR and 30-day mortality and length of stay [32]. Qiu et al. used data from the MIMIC-IV database to explore the relationship between RAR and hospital mortality in COPD patients admitted to ICU, and they found higher RAR (>5.315) was associated with hospital mortality [31]. However, the previous studies have either included small samples or focused on patients with acute exacerbation of COPD in hospital or even ICU. The patients in research studies cannot represent the overall population of COPD patients. In our study, the patients were from the NHANES database, which represented the American population of all ages. We found that RAR was independently associated with all-cause mortality, especially CLRD mortality of COPD patients, whether considering RAR as a continuous value or stratified by quartiles. HR of Q4 RAR for all-cause mortality of COPD was 2.88, and 3.39 for CLRD mortality. CLRD mortality referred to death from chronic airway diseases such as asthma, emphysema, and bronchitis, which had greater value than all-cause mortality in predicting the mortality risk of COPD. These results suggested that RAR had unique predictive value for mortality risk in COPD.

Although substantial and reliable evidence supports the clinical significance of RDW and albumin in COPD, the underlying mechanism remains unclear. Undoubtedly, aging can contribute to the development of COPD [46,47]. Shortening of telomere length is a hallmark of cellular aging. Kozlitina and Garcia found that shorter telomere lengths were significantly and independently associated with increased RDW [48]. A cross-sectional study showed shorter telomeres were more frequent in erythrocyte macrocytosis [29]. Short telomeres lead to cell senescence of haematopoietic progenitors, especially those of the erythroid lineage, thus leading to increased replicative stress and impaired maturation of the erythroid lineage. It is therefore not unexpected that increased RDW may be significantly associated with aging-related conditions such as COPD. Inflammation is closely associated with COPD and its comorbid conditions [49–51]. Albumin has outstanding capacity to bind pro-inflammatory substances and mediators of inflammation, as well as participating in antigen presentation [27]. Lippi’s retrospective analysis showed that erythrocyte sedimentation rate and high sensitivity C-reactive protein increased significantly when RDW increased [24]. Inflammation might promote anisocytosis through impairment of iron metabolism and disruption of response to erythropoietin [52]. Inflammation can also lower erythrocyte survival, thus leading to a more mixed population of erythrocytes in the circulation [23]. COPD can also be attributed to oxidative stress [51,53]. Serum albumin defends against extra- and intracellular oxidative stress [22,26,28]. Malnutrition in COPD is associated with impaired lung function, increased hospitalization, and increased mortality [28,54,55]. Nutritional deficiencies can cause an increase in RDW and a decrease in albumin. Albumin, as an important nutrient, maintains appropriate plasma osmotic pressure, and serves as a carrier for endogenous bioactive substances and exogenous drug molecules [27]. A prospective study involving 2,465 patients in Switzerland revealed that elevated C-reactive protein and increased malnutrition risk were associated with low serum albumin levels [14]. Hypoxemia is feature of COPD, which can stimulate a significant increase in serum erythropoietin, thereby inducing the generation of enlarged erythrocytes, resulting in an increase in RDW [56]. In summary, the potential biological mechanisms behind the association between RDW, albumin, and COPD mortality may involve shorter telomere lengths, inflammation, oxidative stress, malnutrition, and hypoxemia. However, it is still unclear whether elevated RAR is a result of severe COPD or a cause that promotes the occurrence and development of COPD. Basic experiments involving experimental animals or cells are needed to explore the underlying biological connections.

There were several limitations to this study. First, this observational study could only investigate the association between RAR and mortality of COPD patients. Although the relationship between RAR and all-cause and CLRD mortality was revealed, no causal relationship could be obtained. Second, a single examination of RDW and albumin at recruitment ignored the impact of dynamic changes in RAR. An increase or decrease in RAR compared to baseline may better predict the outcome of COPD patients. Third, COPD patients were identified by positive response to a questionnaire, specific medication history, and FEV1/FVC ratio < 0.7 in this study, which differs from FEV1/FVC ratio < 0.7 post-bronchodilation. However, our approach is consistent with methods effectively used in numerous published studies [2,25,33–38]. According to the recent GLOD report, some individuals may exhibit respiratory symptoms and/or structural lung lesions (e.g., emphysema) and/or physiological abnormalities (including low FEV1, gas trapping, hyperinflation, reduced lung diffusing capacity and/or rapid FEV1 decline) without demonstrating airflow obstruction (FEV1/FVC ≥ 0.7 post-bronchodilation), and are therefore labelled ‘Pre-COPD’. Subjects with Pre-COPD are at risk of developing airflow obstruction over time. Some participants in our study may fall under the Pre-COPD category, who also deserve attention. We have not excluded this group of patients and hope that this will raise awareness of the Pre-COPD condition.

Despite all this, the recent work is the first to investigate the relationship between RAR and CLRD mortality / all-cause mortality in the overall population of COPD patients, which is a representative sample of American adults. Multivariable regression and subgroup analyses were conducted to control for socio-demographic, dietary, lifestyle factors, comorbidities, and other potential confounders. We found that RAR was independently associated with all-cause mortality and especially CLRD mortality in a dose-response manner in patients with COPD.

## Conclusion

We revealed that RAR was independently connected with all-cause mortality, as well as CLRD mortality in the overall population of COPD patients. HR for mortality of COPD was approximately three-fold higher for Q4 versus Q1 of RAR. RAR was a simple and inexpensive parameter that has potential to be a novel and promising predictor to identify COPD individuals with high mortality risk. Nevertheless, further prospective studies are required to explore the causal relationship between RAR and COPD mortality.
